# Intimate Partner Violence among women living in families with children under the poverty line and its association with common mental disorders during COVID-19 pandemics in Ceará, Brazil

**DOI:** 10.1186/s12889-023-16233-2

**Published:** 2023-07-06

**Authors:** Sâmia GMO Giacomini, Márcia MT Machado, Onélia MML de Santana, Sabrina GMO Rocha, Camila M. de Aquino, Laécia GA Gomes, Lucas S. de Albuquerque, Maria D. A. de Soares, Álvaro JM Leite, Luciano L. Correia, Hermano AL Rocha

**Affiliations:** 1 Department of Community Health, Federal University of Ceará, Fortaleza, CE Brazil; 2 Laboratory of Epidemiology and Data Analysis, University Health Center ABC. FMABC, Santo André, São Paulo, Brazil; 3 Department of Maternal and Child Health, Federal University of Ceará, Fortaleza, CE Brazil; 4 Social Protection Secretariat. Ceará State Government, Fortaleza, CE Brazil

**Keywords:** Intimate Partner violence, COVID-19, Mental Disorders, Family Conflict

## Abstract

**Background:**

Intimate partner violence (IPV) is a pervasive public health issue that affects millions of women worldwide. Women living below the poverty line experience higher rates of violence and fewer resources to escape or cope with the abuse, and the COVID-19 pandemic has significantly impacted women’s economic well-being worldwide. We conducted a cross-sectional study in Ceará, Brazil, on women in families with children living below the poverty line at the peak of the second wave of COVID to assess the prevalence of IPV and its association with common mental disorders(CMD).

**Methods:**

The study population comprised families with children up to six years of age who participated in the cash transfer program “*Mais Infância*”. The families selected to participate in this program must meet a poverty criterion: families must live in rural areas, in addition to a monthly per capita income of less than US$16.50 per month. We applied specific instruments to evaluate IPV and CMD. To access IPV, we used the Partner Violence Screen (PVS). The Self-Reporting Questionnaire (SRQ-20) was used to assess CMD. To verify the association between IPV and the other evaluated factors with CMD, simple and hierarchical multiple logistic models were used.

**Results:**

Of the 479 participant women, 22% were positively screened for IPV (95% CI 18.2–26.2). After multivariate adjustment, the chances of CMD are 2.32 higher in women exposed to IPV than in those not exposed to IPV ((95%CI 1.30–4.13), p value = 0.004). CMD was also associated with job loss during the COVID-19 pandemic (ORa 2.13 (95% CI 1.09–4.35), p-value 0.029). In addition to these, separate or single marital status, as well as non-presence of the father at home and food insecurity were associated with CMD.

**Conclusion:**

We conclude that the prevalence of intimate partner violence in families with children up to six years of age living below the poverty line in Ceará is high and is associated with greater chances of common mental disorders in mothers. Also, job loss and reduced access to food caused by the Covid 19 pandemic exacerbated both phenomena, constituting a double burden generator factor on mothers.

**Supplementary Information:**

The online version contains supplementary material available at 10.1186/s12889-023-16233-2.

## Introduction

Intimate partner violence (IPV) is a pervasive public health issue that affects millions of women worldwide. According to the World Health Organization, approximately one in three women globally have experienced physical or sexual IPV or both at some point in their lifetime [1, 2]. IPV is not limited to a specific region or culture but is prevalent across all geographic areas, religions, and socioeconomic backgrounds. IPV is a form of gender-based violence that results in physical, sexual, or psychological harm by a current or former partner. Physical IPV can range from slapping to severe physical assault, while sexual IPV includes acts such as forced sexual activity. Psychological abuse can include threats, intimidation, and controlling behavior. IPV can result in a range of adverse health outcomes, including physical injuries, mental health disorders, and chronic illnesses 3].

The COVID-19 pandemic has significantly impacted women’s economic well-being worldwide, with many experiencing increased financial insecurity and poverty. The pandemic has led to widespread job losses and decreased economic activity, particularly in industries where women are overrepresented, such as hospitality and retail [4]. Additionally, women are more likely than men to work in informal or precarious employment, which is more vulnerable to the economic effects of the pandemic. Women have also shouldered a disproportionate burden of unpaid care work, including childcare and household duties, which has increased during the pandemic due to school closures and lockdowns [5]. This has led to many women leaving the workforce to care for children or family members, further exacerbating financial insecurity and poverty.

IPV is a significant issue in Brazil, affecting many women across the country. Brazil has one of the highest rates of violence against women globally, with IPV being a common form of violence [6]. According to a study conducted by the Brazilian Institute of Geography and Statistics, 28.9% of women in Brazil (18 million of women) have reported experiencing some form of IPV at some point in their lives [6]. In addition, the COVID-19 pandemic has had a significant impact on Brazil, resulting in a high number of cases and deaths. As of March 2023, Brazil has reported over 27 million confirmed cases and over 870,000 deaths due to COVID-19, making it the second-highest country in terms of the total number of cases and deaths worldwide [7].

The increase in poverty during pandemics among women is a major issue. Women living below the poverty line experience higher rates of violence and fewer resources to escape or cope with the abuse [8]. Poverty is often linked to factors that increase the risk of IPV, including social exclusion, limited access to education and employment, and living in unsafe environments [9]. These factors can contribute to a lack of agency and control over one’s life, making it more difficult to leave an abusive relationship or seek help [10]. Research suggests that women living below the poverty line are at a higher risk of experiencing IPV than those living above the poverty line [11]. In the United States, for example, women living in poverty experience IPV at a rate five times higher than that of women who do not live in poverty [12]. Additionally, women who experience poverty and IPV are more likely to experience chronic health conditions, including mental health disorders, than women who experience IPV alone.

Mental health disorders are more prevalent among survivors of IPV. IPV is a significant risk factor for the development of common mental disorders, including depression, anxiety, and post-traumatic stress disorder (PTSD) [13]. Studies have found a significant association between IPV and depression, with women who experience IPV being twice as likely to develop depression compared to women who have not experienced violence [14]. In addition to the IPV burden on mental health, the COVID-19 pandemic fueled a mental health deterioration in women [15].

IPV is highly prevalent worldwide, and even more so in developing countries and in poor women. The COVID-19 pandemic has disproportionately increased poverty among women, which may have led to an increase in IPV, in addition to deteriorating women’s mental health, which is negatively affected by IPV. However, surveys of women living in extreme poverty on the occurrence of IPV and their mental status are exceedingly rare. There has been no robust recent research in this field during the pandemic in the developing world. Therefore, we aimed to describe the prevalence of IPV in the population of women in the state of Ceará, as well as the factors associated with this phenomenon, and also to investigate the association between IPV and CMD in women.

## Methods

### Design

A cross-sectional study was carried out through telephone contact from May to July 2021, during the peak of the second wave of the COVID-19 pandemic in Ceará, a state in Northeastern Brazil.

### Setting and sample

Ceará is a poor state in Northeastern Brazil, with an average per capita income of US$150.00. Subsistence farming is the predominant economic activity in the state’s rural areas, although commerce has become very important in Ceará’s economy, making up more than 70% of the state’s GDP. The state’s estimated population for 2021 was 9.2 million inhabitants, according to the Brazilian Institute of Geography and Statistics (IBGE), making it the eighth most populous state in the country.

The study population comprised families with children up to six years of age who participated in the cash transfer program ‘*Mais Infância’* (More Childhood) in 2021. The families selected to participate in this program must meet three poverty criteria: houses whose walls are made of suboptimal materials (wattle and daub, straw, reclaimed wood) without bathrooms or sanitation and no running water in at least one room, (and families should live in rural areas), in addition to a monthly *per capita* income of less than US$16.50. Apart from the State cash transfer program, these families also receive the Brazilian federal government’s conditional cash transfer program (*Bolsa Família*). This population is listed in the records of the government of the state of Ceará, which have the address and telephone numbers of all families that fulfill these criteria, a total of 48,000 families.

This list was used to randomly select 500 families for study participation. This number was obtained considering events with a prevalence of 25%, the prevalence of IPV estimated for Brazil in previous studies, a type 1 error of 5%, and a precision of 5%, reaching an estimated 288 participants at minimum. To compensate for possible losses, we selected almost twice the minimum number.

### Data collection

Interviews were carried out by researchers who were trained explicitly for this purpose by the research coordination team, using a standardized electronic form to prevent possible input errors. In case of failure to contact a study participant after up to three attempts on three different days, the researchers attempted to contact the commercial establishments (such as grocery stores) close to the desired addresses in a last effort to reach the sampled participants. The interviewers were not part of the cash transfer program visitors.

### Variables

Initially, a sociodemographic questionnaire was applied. The analyzed variables comprised: gender, ethnicity, age, family income, marital status, work, relationship with the child’s father, religion, zone of living (if rural or urban), housing conditions, smoking status and presence of physical impairment. All these variables were self-reported by the interviewed women. Additionally, we obtained information about changes in life of women due to pandemics, notedly job loss, food shortage, and emergency aid from the government.

Then, we applied specific instruments to evaluate IPV, CMD, and food insecurity. To access IPV, we used the Partner Violence Screen (PVS). This scale consists of 3 questions, namely:

1. Have you been hit, kicked, punched, or otherwise hurt by someone within the past year? If so, by whom?
2. Do you feel safe in your current relationship?
3. Is there a partner from a previous relationship who is making you feel unsafe now?

We considered that a positive response to any 1 of the three questions constitutes a positive screen for partner violence [1].

Food insecurity was assessed using the Brazilian Food Insecurity Scale (EBIA, *Escala Brasileira de Insegurança Alimentar*), which has been validated in Brazil for food security screening and recommended by the Brazilian Ministry of Social Development and Fight against Hunger. In this study, we used the short version of the EBIA, which contains five questions, of which answers vary from never having experienced the measured aspect of food insecurity to experiencing it every day [16]. The Self-Reporting Questionnaire (SRQ-20) assessed maternal common mental disorders. The SRQ-20 has been validated in several countries, including Brazil [17]. Each SRQ-20 affirmative answer scores a value of 1 to constitute the final score by summing up all answers to all 20 questions. The scores obtained are related to the probability of nonpsychotic disorder, ranging from 0 (no probability) to 20 (extreme probability). In our analysis, cases with scores equal to or greater than eight were considered positive [18].

### Statistical analysis

Descriptive statistics and prevalence rates of positive screening for IPV are presented. The association of the evaluated factor with IPV was assessed using the chi-square test for categorical variables and the Mann-Whitney test for continuous variables. Kolmogorov-Smirnov normality tests were performed for numerical variables. To verify the association between IPV and the other evaluated factors with CMD, simple and multiple logistic models were used. The factors used as potential confounders of CMD were chosen based on our previous research. In the multiple regressive models, the variables that presented p less than 0.05 in the simple regressive models were performed in a hierarchical way, classifying the selected factors between distal and proximal of the CMD outcome. At proximal level we included the variables IPV, Maternal age, and Maternal education; at distal level, we included the variables Marital status, Food insecurity, Change in food availability for your family after COVID-19 pandemic, Job loss during the COVID-19 pandemic, The father of the child under 6 years old lives in the same house, The child’s father sometimes sees or stays with the child, and Smoking The data obtained in the collection were tabulated and analyzed using the IBM SPSS Statistics for Windows software, Version 23.0. Armonk, NY: IBM Corp. IBM Corp. Released 2015.

### Ethical aspects

This project was submitted to the Research Ethics Committee (CEP) of Universidade Federal do Ceará under number CAAE 42815720.4.0000.5054 and was approved according to opinion number 4.565.697. Free and informed consent was obtained from all research participants, and these were recorded on the online platform and written during the in-person visits.

## Results

In total, 479 women participated in the study, and 415 answered questions about IPV. Of those who responded, 22% were positively screened for IPV (95% CI 18.2–26.2). IPV was also associated with CMD (p-value < 0.001). Supplementary Table 1 presents the results of the prevalence of responses for each of the items on the scale, as well as for their possible combinations. (supp Table 1) IPV was more frequent in women exposed to food insecurity (30.6% vs. 18.6%, p-value 0.007) and in women whose father of their children was not present at home (34% vs. 15.5%, p-value < 0.001) and did not even occasionally visit the child (41.7% vs. 30.4%, p-value < 0.001). Furthermore, job loss due to the COVID-19 pandemic was associated with higher IPV, with almost twice as many women who lost their jobs reporting IPV compared to those who kept their jobs (38.5% vs. 20.2%, p-value 0.005). All characteristics can be found in Table 1 (Table 1). Supplementary Table 2 presents the results of the sensitivity analysis for the IPV outcome constructed only with items 2 and 3 of the scale. (supp Table 2)

**Table 1:** Characteristics of the evaluated sample and the bivariate relationship of positive screening factors for IPV.

	IPV positive (group prevalence)*	Total	p-value
Housing Zone			0.523
Rural	65 (21.4)	304	
Urban	27 (24.3)	111	
Maternal Age (years, mean)	31.9 (9.3)	31.1 (7.2)	0.611
Child’s Age (months, mean)	44.2 (15.3)	42.7 (16.7)	0.476
Maternal education			0.117
Up to 8 years	35 (18.3)	191	
More than 8 years	53 (24.8)	214	
Marital Status			0.094
Married	14 (16.1)	87	
Separate	7 (25.9)	27	
Single	35 (30.2)	116	
Stable Union	7 (17.9)	39	
Widow	2 (50.0)	4	
Lives with partner	27 (19.0)	142	
Ethnicity			0.885
Asian	1 (33.3)	3	
White	10 (20.8)	48	
Brown	76 (22.4)	340	
Black	5 (23.8)	21	
Religion			0.658
Catholic	58 (20.9)	277	
None	7 (33.3)	21	
Protestant/Evangelical	26 (23.0)	113	
Umbanda/Candomblé	1 (50.0)	2	
Monthly family income in *reais (mean)*	551.3 (243.12)	527.4 (227.1)	0.056
Food insecurity			**0.007**
Yes	38 (30.6)	124	
No	54 (18.6)	291	
Availability of internet at home			0.685
No	36 (21.2)	170	
Yes	56 (22.9)	245	
Participation in any activity developed at the government social assistance center			0.241
No	80 (21.1)	379	
Yes	11 (33.3)	33	
Change in food availability for your family after COVID-19 pandemic			0.823
Has not changed	14 (20.0)	70	
Yes, it increased	5 (26.3)	19	
Yes, it decreased	73 (22.4)	326	
Job loss during the COVID-19 pandemic			**0.005**
Yes	20 (38.5)	52	
No	72 (20.2)	356	
Receiving governmental emergency aid			0.345
No	4 (44.3)	12	
Yes	88 (21.8)	403	
Her child’s father lives at home			**< 0.001**
No	51 (34.0)	150	
Yes	41 (15.5)	265	
Her child’s father sometimes stays with the child			**< 0.001**
No	20 (41.7)	48	
Yes	31 (30.4)	102	
Current maternal work			0.310
Yes, at home	8 (34.8)	23	
Yes, for selling away from home	12 (24.5)	49	
No, only at home (housework)	72 (21.2)	339	
Smoking			0.056
No	85 (21.5)	396	
Yes	7 (41.2)	17	
Common Mental Disorder			**< 0.001**
No	54 (17.4)	310	
Yes	38 (36.2)	105	

*Numbers are n(%) or Mean (Standard Deviation)

**Fig. 1:**
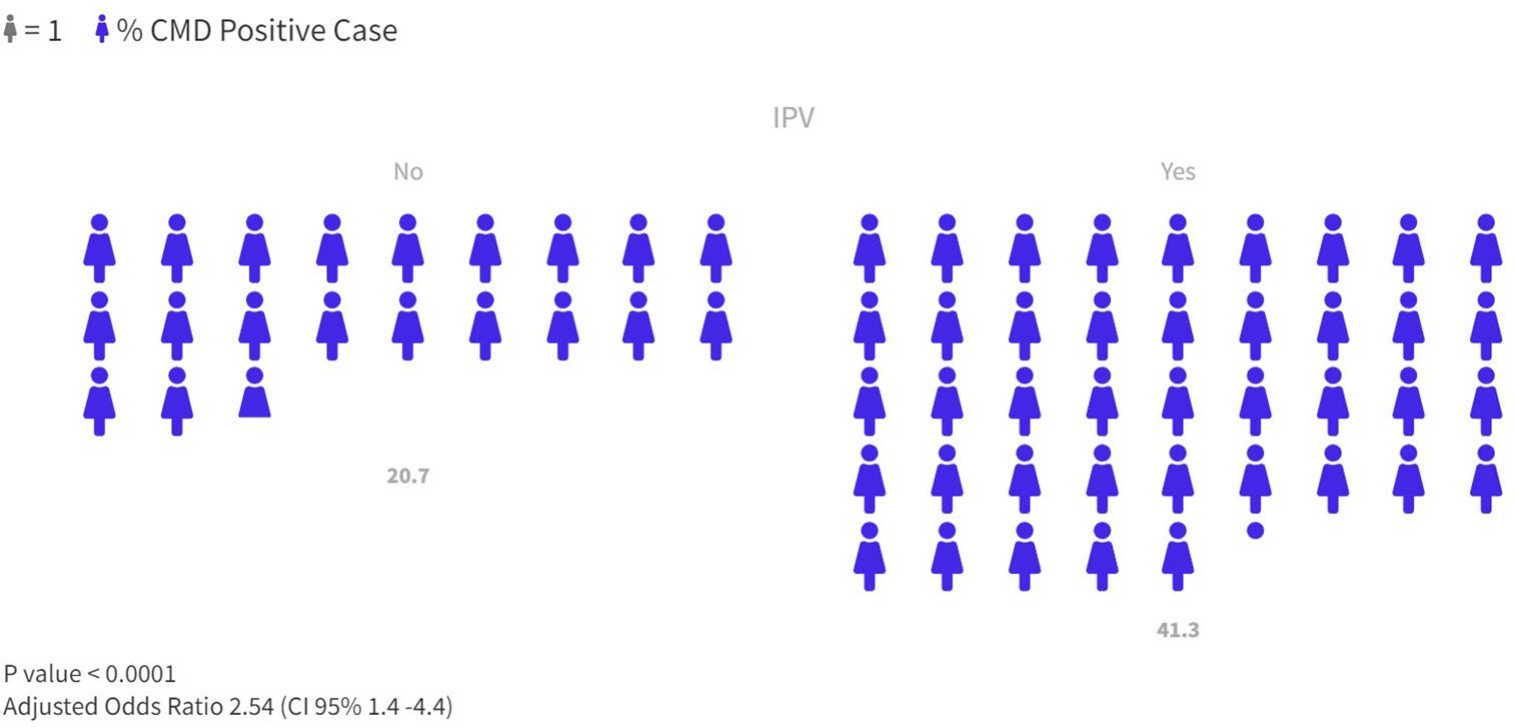
Prevalence of positive CMD in groups with positive and negative IPV screening. IPV: Intimate Partner Violence

In total, 21.9% of respondents were positively screened by the SRQ as having CMD. Among the women who reported IPV, the prevalence of CMD was 41.3%, while among those who did not, it was 20.7% (OR 2.7 [CI95% 1.6–4.4], p-value < 0.001), as can be seen in Fig. 1.

In Table 2, the factors associated with CMD are explored. Higher maternal age was associated with higher odds of CMD (p = 0.032), and higher maternal education was protective of CMD (p = 0.029). Separated or single women were more likely to have CMD compared to women who lived with partners (OR 2.92 [1.22–7.00], p = 0.016; OR 2.15 [1.22–3.81], p-value 0.008, respectively). Food insecurity was a risk factor for CMD, as well as the decrease in available food during the COVID-19 pandemic. Job loss due to the pandemic has also led to higher prevalence of CMD among women. The absence of the child’s father was also associated with CMD among women, with higher odds of CMD for women who did not live with the child’s fathers (OR 1.81 [1. 15–2.84], p = 0.010), and for those where the child’s father did not occasionally visit (OR 3.08 [1.49–6.35], p-value = 0.002). Furthermore, all individual IPV scale items were associated with CMD. The complete data can be seen in Table 2.

**Table 2:** Factors associated with positive screening for CMD in the evaluated sample

	CMD Positive*	Total	OR (CI 95%)	P-value
Housing zone				0.130
Rural	71 (23.4)	304	0.69 (0.42–1.11)	
Urban	34 (30.6)	111	1	
Maternal age	32.5 (7.8)	31.1 (7.2)	1.03 (1.00–1.06)	**0.032**
Child’s age				
	45.3 (15.0)	42.7 (16.7)	1.01 (0.99–1.02)	0.063
Maternal education				**0.029**
More than 8 years	39 (20.4)	191	0.60 (0.38–0.95)	
Up to 8 years	64 (29.9)	214	1	
Marital status				**0.023**
Married	17 (19.5)	87	1.03 (0.52–2.0)	0.922
Separate	11 (40.7)	27	2.92 (1.22–7.00)	**0.016**
Single	39 (33.6)	116	2.15 (1.22–3.81)	**0.008**
Stable union	9 (23.1)	39	1.27 (0.54–3.00)	0.544
Widow	2 (50.0)	4	4.2 (0.57–31.60)	0.574
Lives with partner	27 (19.0)	142	1	
Ethnicity				0.368
Asian	2 (66.7)	3	6.4 (0.47–86.34)	0.162
White	12 (25.0)	48	1.06 (0.32–3.53)	0.916
Indigenous	2 (66.7)	3	6.40 (0.47–86.34)	0.162
Brown	84 (24.7)	340	1.05 (0.37–2.95)	0.926
Black	5 (23.8)	21	1	
Monthly family income in *reais*				
	514.9 (190.9)	527.4 (227.1)	1.00 (0.99–1.00)	0.512
Food insecurity				**< 0.001**
Yes	50 (40.3)	124	2.94 (1.85–4.76)	
No	55 (19.8)	291	1	
Number of inhabitants of the house				0.16
	4 (2)	3 (2)	1.10 (0.96–1.26)	
Availability of internet at home				0.816
No	42 (24.7)	170	0.94 (0.60–1.48)	
Yes	63 (25.7)	245	1	
Change in food availability for your family after COVID-19 pandemic				**0.026**
Yes, it decreased	89 (27.3)	326	2.56 (1.22–5.56)	**0.013**
Yes, it increased	7 (36.8)	19	1.55 (0.59–4.07)	0.37
Has not changed	9 (12.9)	70	1	
Job loss during the COVID pandemic				
Yes, I lost informal employment	22 (42.3)	52	2.33 (1.32–4.17)	**0.004**
No, no longer working	81 (22.8)	356	1	
Receiving emergency aid from the federal government for the pandemic				0.981
No	3 (25.0)	12	0.98 (0.26–3.70)	
Yes	102 (25.3)	403	1	
The father of the child under 6 years old lives in the same house				**0.010**
No	49 (32.7)	150	1.81 (1.15–2.84)	
Yes	56 (21.1)	265	1	
The child’s father sometimes sees or stays with the child				**0.002**
No	24 (50.0)	48	3.08 (1.49–6.35)	
Yes	25 (24.5)	102	1	
Current maternal work				0.912
Does not work at all (not even housework)	1 (25.0)	4	0.98 (0.1–9.55)	0.987
Yes, at home, out	7 (30.4)	23	1.28 (0.51–3.23)	0.591
Yes, away from home	11 (22.4)	49	0.85 (0.41–1.74)	0.659
Yes, only at home (housework)	86 (25.4)	339	1	
Smoking				**0.044**
Yes	8 (47.1)	17	2.78 (1.03–7.69)	
No	97 (24.5)	396	1	
Do you feel safe in your current relationship?				**0.010**
No	14 (45.2)	31	2.70 (1.27–5.88)	
Yes	91 (23.7)	384	1	
Have you been hit, kicked, punched, or otherwise hurt by someone within the past year?				**< 0.001**
Yes	14 (60.9)	23	5.26 (2.17–12.5)	
No	91 (23.2)	392	1	
Is there a partner from a previous relationship who is making you feel unsafe now?				**< 0.001**
Yes	30 (50)	60	3.85 (2.13–6.67)	
No	75 (21.1)	355	1	

*Numbers are n(%) or Mean (Standard Deviation)

The results of the multivariate model of the association between IPV and CMD with the inclusion of the significant factors from the previous step can be seen in Table 3. The chances of CMD are 2.32 higher in women exposed to IPV than in those not exposed to IPV (CI95% 1.30–4.13), p = 0.004. CMD was also associated with job loss during the COVID-19 pandemic (OR_a_ 2.13 [95%CI 1.09–4.35], p = 0.029). In addition to these, separate or single marital status and non-presence of the father at home, and food insecurity were also independently associated with CMD. (Table 3) Supplementary Table 3 presents the results of the sensitivity analysis for the IPV outcome constructed only with items 2 and 3 of the scale. (supp Table 3).

**Table 3:** Factors associated with increased odds of CMD after multivariate adjustment

	OR (CI 95%)	P-value	AOR (CI 95%)	P-value adj
*Proximal level*				
IPV		**< 0.001**		**0.004**
Yes	2.68 (1.64–4.40)		2.32 (1.30–4.13)	
No	1		1	
Maternal age	1.03 (1.00–1.06)	**0.032**	1.02 (0.98–1.05)	0.278
Maternal education		**0.029**		0.173
More than 8 years	0.60 (0.38–0.95)		1.44 (0.85–2.47)	
Up to 8 years	1		1	
*Distal Level*				
Marital status		**0.023**		0.073
Married	1.03 (0.52–2.0)	0.922	1.11 (0.53–2.31)	0.773
Separate	2.92 (1.22–7.00)	**0.016**	5.98 (1.64–21.82)	**0.007**
Single	2.15 (1.22–3.81)	**0.008**	4.02 (1.57–10.33)	**0.004**
Stable union	1.27 (0.54–3.00)	0.544	1.45 (0.58–3.65)	0.42
Widow	4.2 (0.57–31.60)	0.574	5.07 (0.49–52.36)	0.172
Lives with partner	1		1	
Food insecurity		**< 0.001**		**0.003**
Yes	2.94 (1.85–4.76)		2.27 (1.33–3.85)	Yes
No	1		1	No
Change in food availability for your family after COVID-19 pandemic		**0.026**		0.14
Has not changed	0.39 (0.18–0.82)	**0.013**	0.56 (0.24–1.26)	0.163
Yes, it increased	1.55 (0.59–4.07)	0.37	2.03 (0.67–6.16)	0.207
Yes, it decreased	1			
Job loss during the COVID-19 pandemic		**0.004**		**0.029**
Yes, I lost informal employment	2.33 (1.32–4.17)		2.13 (1.09–4.35)	
No, not working before pandemics	1		1	
The father of the child under 6 years old lives in the same house		**0.010**		**0.012**
No	1.81 (1.15–2.84)		3.57 (1.33–10.00)	
Yes	1		1	
The child’s father sometimes sees or stays with the child		**0.002**		**0.006**
No	3.08 (1.49–6.35)		3.12 (1.39–7.00)	
Yes	1		1	
Smoking		**0.044**		0.225
No	0.36 (0.13–0.97)		0.48 (0.15–1.55)	
Yes	1		1	

## Discussion

In this study, we identified that IPV prevalence in women living below the poverty line in Ceará is 22% (CI 95% 18.2–26.2), and that factors related to the COVID-19 pandemic, such as women that had job loss, were associated with a higher prevalence of IPV. Furthermore, IPV increased women’s odds of experiencing CMD, regardless of pandemic-associated and other factors.

The prevalence of IPV found in our study is consistent with that identified by other studies in vulnerable locations worldwide. The estimated lifetime prevalence of intimate partner violence is as high as 27% [23–31]% for women between the ages of 15 and 49 years [19]. In Brazil, a study in the southeast of the country estimated lifetime IPV at 21% [20]. The estimated rate in a global study for Southern Latin America was 25%, and these data show that the third highest global prevalence of IPV is in the Andean Latin America region [19]. In fact, a study that specifically evaluated IPV during pregnancy in Peru found a prevalence of 44%, being 21% during pregnancy [21]. On the other hand, the prevalence of women who experienced violence in the last year, closer to what we evaluated in the present study, was estimated at 13% (10–16%) [19], which makes our sample with a much higher prevalence of the phenomenon.

There is a strong association between IPV and poverty. Poverty is a complex and multifaceted phenomenon that encompasses various factors, including income, education, employment, and housing. These factors can affect the risk of IPV by increasing stress levels, limiting access to resources and services, and exacerbating existing inequalities and power imbalances [22]. Several studies have examined the relationship between poverty and IPV. For instance, a study conducted in the United States found that low-income women were more likely to experience IPV than women with higher incomes [22]. Another study conducted in India found that women from poor households were more likely to experience IPV than women from wealthier households [23]. Research also suggests that poverty can have an intergenerational impact on IPV. A study conducted in Canada found that children who grew up in poverty were more likely to experience IPV as adults [24]. This finding highlights the importance of addressing poverty as a key factor in preventing IPV and promoting healthy relationships. Various mechanisms can explain the association between poverty and IPV. Poverty can increase stress levels, which can lead to conflict and violence within relationships. Poverty can also limit access to resources and services, such as healthcare and social support, exacerbating IPV’s negative consequences. Moreover, poverty can exacerbate existing inequalities and power imbalances within relationships, leading to an increased risk of IPV [25]. Women who are economically dependent on their partners may be less likely to leave abusive relationships due to financial constraints, perpetuating the cycle of violence.

The association of IPV with CMD in other populations has been well established [26, 27], and we bring new evidence of this association during the COVID-19 pandemic in families below the poverty line. Research suggests that the association between IPV and CMDs is bidirectional. That is, women with pre-existing mental health conditions may be more vulnerable to experiencing IPV, while experiencing IPV may exacerbate or trigger CMDs [28]. For instance, a longitudinal study conducted in the United States found that women who experienced IPV were more likely to develop symptoms of depression and anxiety over time [29]. A meta-analysis of 41 studies conducted across 22 countries found that women who experienced IPV were more likely to have symptoms of depression, anxiety, and post-traumatic stress disorder (PTSD) than women who did not experience IPV [30]. The mechanisms underlying the association between IPV and CMDs are complex and multifaceted. IPV can lead to psychological trauma, which can contribute to the development of CMDs. Moreover, IPV can disrupt social support networks and limit access to resources and services, exacerbating the negative consequences of CMDs [31]. Furthermore, the impact of IPV on mental health can be compounded by the social and cultural stigma surrounding IPV. Women who experience IPV may feel isolated and ashamed, which can further exacerbate symptoms of CMDs [31]. Furthermore, the experience of IPV can lead to chronic stress, which has been linked to the development of CMD. The chronic stress associated with IPV can activate the hypothalamic-pituitary-adrenal (HPA) axis and increase levels of cortisol, a hormone that has been associated with depression and anxiety [32].

The COVID-19 pandemic has led to negative economic consequences for the population, such as job losses and increased food insecurity [33, 34]. The pandemic has caused a significant economic downturn, leading to widespread job losses and financial insecurity. This economic stress may exacerbate the negative consequences of IPV and CMDs, which can lead to further job loss. A study conducted in Italy suggested that women who experienced job loss during the pandemic were likelier to report IPV than those who did not experience job loss [35]. Similarly, a study conducted in the United States found that job loss due to the pandemic was associated with an increased risk of IPV perpetration and victimization [36]. Moreover, job loss due to the pandemic may exacerbate CMD. The economic stress of job loss can lead to feelings of hopelessness, anxiety, and depression, which can exacerbate pre-existing mental health conditions. A study conducted in the USA found that job loss due to the pandemic was associated with an increased risk of CMD, particularly depression and anxiety [37]. The link between job loss due to the pandemic, IPV, CMD, and their negative consequences can form a vicious cycle. For example, job loss may lead to financial insecurity, exacerbating the negative consequences of IPV and CMD, leading to further job loss due to decreased productivity, absenteeism, and job turnover.

We also observed that stable marital ties and the presence of the child’s father at home were associated with lower chances of IPV and CMD. The instability in marital relationships can be due to several factors, including communication problems, financial stress, and infidelity [38]. These issues can lead to feelings of anger, jealousy, and resentment, which can contribute to IPV perpetration and victimization. A study conducted in Oregon found that men who reported high levels of marital dissatisfaction were more likely to perpetrate IPV [39]. Moreover, unstable marital relationships may exacerbate the negative consequences of IPV. Women who experience IPV in unstable marital relationships may feel trapped and unable to leave, leading to hopelessness and depression. A study conducted in California found that women who experienced IPV in unstable marital relationships reported higher levels of depression and anxiety compared to those in stable marital relationships [40].

This study had some limitations. First, as this is a cross-sectional study, the associated factors we found cannot be defined as causal. Furthermore, although we used validated IPV and CMD scales, they are not exhaustive of the occurrence of self-efficacy in each individual, even though it has shown good accuracy in the studies that tested it, and the third item on the scale used is different from the scale used by the WHO. Our study found evidence that the absence of the child’s father is associated with common mental disorders in the mother, which may be correlated with a lack of support in the family routine or financial support. However, our study contains information only regarding the absence or presence of the child’s father. Other studies should evaluate the factors correlated with the absence of a partner to better clarify this association and how having or not having the child’s father present contributes to the incidence of common mental disorders in mothers. Furthermore, the use of odds ratios may have overestimated the magnitude of the associations found. Finally, participants who did not answer the phone and were not included in the sample may have biased the result; however, we believe that the prevalence of IPV is higher in non-participants, given that a higher level of poverty must have had an impact on non-participation, which would maintain the relevance of the findings presented here.

## Conclusions

We conclude that the prevalence of intimate partner violence in families with children up to six years living below the poverty line in Ceará is high and is associated with greater chances of common mental disorders in mothers. Job loss and reduced access to food caused by the COVID-19 pandemic exacerbated both phenomena, constituting a double burden factor on mothers. Still, the stable marital bond and the father’s presence at home were protective against IPV and CMD. This evidence can be used for formulating public policies that address these problems.

## Electronic supplementary material

Below is the link to the electronic supplementary material.

Supplementary Material 1

## Data Availability

The datasets used and/or analyzed during the current study are available from the corresponding author upon reasonable request.

## List of Abbreviations

IPV: Intimate Partner Violence
CMD: Common Mental Disorders
