# Supplementary material for: Intimate Partner Violence among women living in families with children under the poverty line and its association with common mental disorders during COVID-19 pandemics in Ceará, Brazil
Source: BMC Public Health. 2023 Jul 6;23:1299. doi: 10.1186/s12889-023-16233-2 (PMC10327360; doi:10.1186/s12889-023-16233-2)
Supplement: Supplementary file 1 — Supplementary Material 1 [file 12889_2023_16233_MOESM1_ESM.docx]

Supplementary Table 1. Characteristics of the evaluated sample and the bivariate relationship of positive screening factors for IPV*.*

| Items from Partner Violence Screen | N | % |
| --- | --- | --- |
| No item | 323 | 77,8 |
| Item 1: Have you been hit, kicked, punched, or otherwise hurt by someone within the past year? | 12 | 2,9 |
| Item 2: Do you feel safe in your current relationship? | 19 | 4,6 |
| Item 3: Is there a partner from a previous relationship who is making you feel unsafe now? | 46 | 11,1 |
| Item 1 + Item 2 | 1 | 0,2 |
| Item 1 + Item 3 | 3 | 0,7 |
| Item 2 + Item 3 | 4 | 1,0 |
| All itens | 7 | 1,7 |

Supplementary Table 2. Characteristics of the evaluated sample and the bivariate relationship of positive screening factors for IPV (sensitivity analysis)*.*

|  | IPV positive (group prevalence)* | Total | p-value |
| --- | --- | --- | --- |
| Housing Zone |  |  | 0.464 |
| Rural | 56 (18.4) | 304 |  |
| Urban | 24 (21.6) | 111 |  |
| Maternal Age (years, mean) | 31.8 (9.6) | 31.2 (7.3) | 0.838 |
| Child's Age (months, mean) | 44.4 (15.8) | 42.7 (16.8) | 0.373 |
| Maternal education |  |  | 0.178 |
| Up to 8 years | 31 (16.2) | 191 |  |
| More than 8 years | 46 (21.5) | 214 |  |
| Marital Status |  |  | 0.006 |
| Married | 10 (11.5) | 87 |  |
| Separate | 7 (25.9) | 27 |  |
| Single | 34 (29.3) | 116 |  |
| Stable Union | 6 (15.4) | 39 |  |
| Widow | 2 (50.0) | 4 |  |
| Lives with partner | 21 (14.8) | 142 |  |
| Ethnicity |  |  | 0.811 |
| Asian | 1 (33.3) | 3 |  |
| White | 8 (16.7) | 48 |  |
| Brown | 66 (19.4) | 340 |  |
| Black | 5 (23.8) | 21 |  |
| Religion |  |  | 0.629 |
| Catholic | 49 (17.7) | 277 |  |
| None | 7 (33.3) | 21 |  |
| Protestant/Evangelical | 23 (20.4) | 113 |  |
| Umbanda/Candomblé | 1 (50.0) | 2 |  |
| Monthly family income in *reais (mean)* | 541.7 (248.7) | 527.5 (227.1) | 0.214 |
| Food insecurity |  |  | **0.006** |
| Yes | 34 (27.4) | 124 |  |
| No | 46 (15.8) | 291 |  |
| Availability of internet at home |  |  | 0.340 |
| No | 29 (17.1) | 170 |  |
| Yes | 51 (20.8) | 245 |  |
| Participation in any activity developed at the government social assistance center | | | 0.099 |
| No | 70 (18.5) | 379 |  |
| Yes | 10 (30.3) | 33 |  |
| Change in food availability for your family after COVID-19 pandemic | | | 0.906 |
| Has not changed | 13 (18.6) | 70 |  |
| Yes, it increased | 3 (15.8) | 19 |  |
| Yes, it decreased | 64 (19.6) | 326 |  |
| Job loss during the COVID-19 pandemic |  |  | **0.002** |
| Yes | 19 (36.5) | 52 |  |
| No | 61 (17.1) | 356 |  |
| Receiving governmental emergency aid | | | 0.610 |
| No | 3 (25.0) | 12 |  |
| Yes | 77 (19.1) | 403 |  |
| Her child’s father lives at home | |  | **< 0.001** |
| No | 49 (24.7) | 150 |  |
| Yes | 31 (11.7) | 265 |  |
| Her child's father sometimes stays with the child | |  | **< 0.001** |
| No | 20 (41.7) | 48 |  |
| Yes | 29 (28.4) | 102 |  |
| Current maternal work |  |  | 0.401 |
| Yes, at home | 7 (30.4) | 23 |  |
| Yes, for selling away from home | 10 (20.4) | 49 |  |
| No, only at home (housework) | 63 (18.6) | 339 |  |
| Smoking |  |  | 0.090 |
| No | 74 (18.7) | 396 |  |
| Yes | 6 (35.3) | 17 |  |
| Common Mental Disorder |  |  | **< 0.001** |
| No | 47 (15.2) | 310 |  |
| Yes | 33 (31.4) | 105 |  |

*Numbers are n(%) or Mean (Standard Deviation)

Supplementary Table 3. Factors associated with increased odds of CMD after multivariate adjustment (sensitivity analysis).

|  | OR (CI 95%) | P-value | AOR (CI 95%) | P-value adj |
| --- | --- | --- | --- | --- |
| *Proximal level* |  |  |  |  |
| IPV |  | **< 0.001** |  | **0.022** |
| Yes | 2.56 (1.53 - 4.29) |  | 2.31 (1.11 - 3.73) |  |
| No | 1 |  | 1 |  |
| Maternal age | 1.03 (1.00 - 1.06) | **0.032** | 1.02 (0.98 - 1.06) | 0.246 |
| Maternal education |  | **0.029** |  | 0.164 |
| More than 8 years | 0.60 (0.38 - 0.95) |  | 1.46 (0.86 - 2.48) |  |
| Up to 8 years | 1 |  | 1 |  |
| *Distal Level* |  |  |  |  |
| Marital status |  | **0.023** |  | 0.073 |
| Married | 1.03 (0.52 - 2.0) | 0.922 | 1.12 (0.54 - 2.32) | 0.755 |
| Separate | 2.92 (1.22 - 7.00) | **0.016** | 5.48 (1.51 - 19.79) | **0.010** |
| Single | 2.15 (1.22 -3.81) | **0.008** | 3.81 (1.49 - 9.74) | **0.005** |
| Stable union | 1.27 (0.54 - 3.00) | 0.544 | 1.45 (0.58 - 3.63) | 0.423 |
| Widow | 4.2 (0.57 - 31.60) | 0.574 | 4.79 (0.47 - 48.93) | 0.186 |
| Lives with partner | 1 |  | 1 |  |
| Food insecurity |  | **< 0.001** |  | **0.002** |
| Yes | 2.94 (1.85 - 4.76) |  | 2.28 (1.35 - 3.85) | Yes |
| No | 1 |  | 1 | No |
| Change in food availability for your family after COVID-19 pandemic | | **0.026** |  | 0.14 |
| Has not changed | 0.39 (0.18 - 0.82) | **0.013** | 0.58 (0.25 - 1.26) | 0.159 |
| Yes, it increased | 1.55 (0.59 - 4.07) | 0.37 | 2.17 (0.73 - 6.44) | 0.164 |
| Yes, it decreased | 1 |  | 1 |  |
| Job loss during the COVID-19 pandemic | | **0.004** |  | **0.028** |
| Yes, I lost informal employment | 2.33 (1.32 - 4.17) |  | 2.13 (1.09 - 4.17) |  |
| No, not working before pandemics | 1 |  | 1 |  |
| The father of the child under 6 years old lives in the same house |  | **0.010** |  | **0.016** |
| No | 1.81 (1.15 - 2.84) |  | 3.32 (1.25 - 8.85) |  |
| Yes | 1 |  | 1 |  |
| The child's father sometimes sees or stays with the child |  | **0.002** |  | **0.006** |
| No | 3.08 (1.49 - 6.35) |  | 3.07 (1.37 - 6.85) |  |
| Yes | 1 |  | 1 |  |
| Smoking |  | **0.044** |  | 0.202 |
| No | 0.36 (0.13 - 0.97) |  | 0.47 (0.15 - 1.50) |  |
| Yes | 1 |  | 1 |  |
